# MiR-26a-5p enhances cells proliferation, invasion, and apoptosis resistance of fibroblast-like synoviocytes in rheumatoid arthritis by regulating PTEN/PI3K/AKT pathway

**DOI:** 10.1042/BSR20182192

**Published:** 2019-07-26

**Authors:** Zhengping Huang, Shan Xing, Meng Liu, Weiming Deng, Yunqing Wang, Zhixiang Huang, Yukai Huang, Xuechan Huang, Chiwei Wu, Xin Guo, Xia Pan, Jiawei Jiang, Fan Feng, Tianwang Li

**Affiliations:** 1 Department of Rheumatology and Immunology, Guangdong Second Provincial General Hospital, Guangzhou 510317, PR China; 2 Department of Clinical Laboratory, State Key Laboratory of Oncology in South China, Collaborative Innovation Center for Cancer Medicine, Sun Yat-sen University Cancer Center, Guangzhou 510060, PR China; 3 Department of Traumatology, Guangdong Second Provincial General Hospital, Guangzhou 510317, PR China

**Keywords:** fibroblast-like synoviocytes, miR-26a-5p, PTEN, rheumatoid arthritis

## Abstract

Behavior alterations in fibroblast-like synoviocytes (FLS) contribute to a pivotal role in pathogenesis of rheumatoid arthritis (RA). MiRNAs are closely involved in a variety of pathologic conditions. In the present study, we aimed to screen for the aberrant expression of miRNAs in rheumatoid arthritis fibroblast-like synoviocytes (RA-FLS) to further identify the altered expression of miR-26a-5p in RA-FLS and to investigate the role of miR-26a-5p in RA. The altered expression of miR-26a-5p in RA-FLS was screened by microarray analysis and confirmed by quantitative real time PCR. The effect of miR-26a-5p on proliferation, cell cycle, apoptosis, and invasion in RA-FLS were studied. The verification of miR-26a-5p target mRNA and downstream signaling pathway was elucidated by bioinformatics analysis, dual luciferase reporter assay, and western blot. Expression of miR-26a-5p was higher in RA-FLS than in fibroblast-like synoviocytes from osteoarthritis patients and trauma patients. Overexpression of miR-26a-5p RA-FLS promoted cells proliferation, G1/S transition, cells invasion, and resisted apoptosis in RA-FLS, whereas it led to contrary effects when inhibiting the expression of miR-26a-5p. The 3′UTR of tensin homolog (PTEN) was directly targetted by miR-26a-5p and activation of phosphoinositide 3-kinase (PI3K)/AKT pathway was observed when overexpression of miR-26a-5p. Our study suggested that miR-26a-5p has a complementary role in cells proliferation, invasion, and apoptosis of RA-FLS, which may be attributed to its activation effect on PI3K/AKT signaling pathway via targetting PTEN. MiR-26a-5p is likely to be a clinically helpful target for novel therapeutic strategies in RA.

## Introduction

Rheumatoid arthritis (RA) is a common systemic autoimmune disease, characterized by chronic inflammatory of the joints, destruction in cartilage and joint bone, and a series of extra-articular manifestations including rheumatoid nodules, pulmonary interstitial disease, and Felty’s syndrome [1,2]. It is commonly accepted that fibroblast-like synoviocytes (FLS) play a crucial role in the pathological changes of RA [3]. Rheumatoid arthritis FLS (RA-FLS) display a range of aggressive features including hyperproliferation, apoptosis resistance, increased invassiveness, and production of inflammatory mediators [4,5]. Increasing evidence suggests that the interaction of the environmental, immunological, and genetic components contribute to the progression of RA [6]; however, the pathogenesis of RA, in particular the molecular mechanism of behavior alterations in RA-FLS, has remained to be completely clarified.

MiRNAs are a group of endogenous approximately 22-nucleotide-long noncoding RNAs, negatively regulating the expression of protein-coding genes by post-transcriptional regression or degradation of mRNA though targetting 3′UTR of the genes [7]. It is believed that miRNAs potentially regulate at least 20–30% of all human protein coding genes [8], which are closely involved in a series of biological process including cell cycle, cellular proliferation, differentiation, apoptosis, and immunoreaction [9–11]. It has been found that altered expression of miRNAs play functional roles in the development of a variety of human diseases, including cancer [12], gastrointestinal diseases [13], cardiovascular diseases [14], and autoimmune diseases [15]. In particular, several previous studies have focussed on the role of miRNAs in RA-FLS and revealed that up-regulated or down-regulated certain miRNAs were involved in the alteration of proliferation, invasion, and apoptosis in RA-FLS, indicating that the identification of these miRNAs might contribute to our understanding about the molecular mechanism of RA [16–18].

To further investigate the potential role of miRNAs in RA, in the present study we performed microarray analysis to find out the altered expression profile of miRNA in RA-FLS. We found that miR-26a-5p, which plays multiple roles in proliferation and metastasis of various cells [19–21], was up-regulated in RA-FLS, and the finding was confirmed by quantitative real time PCR (qRT PCR). To elucidate the role of miR-26a-5p in RA-FLS, we further studied its effect on cells proliferation, cell cycle, apoptosis, and invasion in RA-FLS, as well as verification of a predicted target and the potential signaling pathway. Finally, our data identified miR-26a-5p as a regulator in cells proliferation, invasion, and apoptosis resistance of RA-FLS, which may be attributed to its activation effect on phosphoinositide 3-kinase (PI3K)/AKT signaling pathway via targetting PTEN. Overall, our findings support a previously undefined function for miR-26a-5p wherein it participates in the activation of RA-FLS, is a potential target for novel therapeutic strategies in RA.

## Materials and methods

### Synovial tissue and fibroblast-like synoviocytes

Synovial tissue specimens were collected during knee joint replacement surgery or synovial biopsy from eight patients with RA, nine patients with OA, and eight patients with joint trauma in Guangdong Second Provincial General Hospital. All RA patients fulfilled the 2010 American College of Rheumatology/European League Against Rheumatism Classification Criteria for Rheumatoid Arthritis [22]. The classical method of enzyme digestion was used to isolate primary fibroblast-like synoviocytes. Synovial tissue specimens were minced and digested with Collagenase Type I (Sigma–Aldrich, U.S.A.) at 37°C for 2 h. After filtration and washing, cells were cultured in high glucose (4500 mg/l) DMEM (Sigma–Aldrich, U.S.A.) with 15% fetal bovine serum (FBS), penicillin (100IU/ml), and streptomycin (100IU/ml). Cells were grown at 37°C in a humidified atmosphere with the presence of 5% CO_2_. The fibroblast-like synoviocytes harvested between passages 4 and 6 were available for the follow-up experiments. Demographic and clinical data of included participants (Han Chinese) are shown in Supplementary Table S1. Ethical approval was granted by the Ethics Committees of Guangdong Second Provincial General Hospital, and the written informed consent was obtained from all the included patients.

### Total RNA extraction

Total RNA was extracted from the fibroblast-like synoviocytes by means of TRIzol reagent (Thermo Scientific, U.S.A.) and the RNA-containing aqueous phase was further purified by RNeasy MinElute Cleanup kit (QIAGEN, Germany) following the manufacturer’s protocols. Total RNA of fibroblast-like synoviocytes was prepared for miRNA expression analyses including microarray analysis and qRT PCR assay. RNA integrity and purity were confirmed with 1% agarose gel electrophoresis and a Nanodrop 2000 spectrophotometer (Thermo Scientific, U.S.A.).

### Microarray analysis

Altered expression of miRNA in RA-FLS and fibroblast-like synoviocytes from knee trauma patients was assessed by microarray analysis. Total RNA samples from three RA patients and three knee joint trauma patients (controls) were labeled with Hy3™ and Hy5™ fluorescent label, respectively, using the miRCURY™ LNA Array power labeling kit (Exiqon, Denmark). Then the samples were hybridized using a miRCURY LNA™ Array microarray kit (Exiqon, Denmark), with capture probes for miRNAs. The hybridization signal was detected by scanner Genepix 4000B (Axon Instruments) and analyzed with Genepix 6.0 (Axon Instruments). Significantly expression of a miRNA between groups was defined as |log2 Ratio |≥1 and *P*<0.05.

### Quantitative real time PCR

qRT-PCR was carried out in all total RNA samples to evaluate miR-26a-5p expression in RA-FLS, OA-FLS, and fibroblast-like synoviocytes from knee trauma patients, using Hairpin-itTM miRNAs qPCR Quantitation kit (GenePharma, China) and Hairpin-itTM U6 snRNA qPCR Normalization kit (GenePharma, China). Small nuclear U6 RNA was quantitated as an internal control for normalization. The quantitative RT-PCR was performed by using PRISM^®^ 7500 Sequence Detection System (ABI, U.S.A.). Quantitative RT-PCR analysis was performed according to manufacture’s instructions. All the tests were repeated three-times. Relative quantitation of miR-26a level was computed by the comparative Ct (2^−ΔΔ*C*^_T_) method. Oligonucleotides sequences used in the qRT-PCR for miR-26a-5p were forward 5′-ACACTCCAGCTGGGTTCAAGTAATCCAGGA-3′, Reverse 5′-TGGTGTCGTGGAGTCG-3′, and for U6 were forward 5′-CTCGCTTCGGCAGCACA-3′, Reverse 5′- ACGCTTCACGAATTTGCGT-3′.

### Transfection

MiR-26a-5p mimic, inhibitor, and corresponding negative control (NC) were synthesized by GenePharma, China. RA-FLS were cultured in DMEM (Sigma–Aldrich, U.S.A.) with 10% FBS for 24 h. Then RA-FLS were transfected by miR-26a-5p mimic, inhibitor, and NC (20 μM) by using lipofectamine 2000 reagent (Invitrogen, U.S.A.) at 60–80% of confluence in well plates. Likewise, cells were grown at 37°C in a humidified atmosphere with the presence of 5% CO_2_. In addition, RA-FLS cells were treated with the PI3K/Akt inhibitor LY294002 (30 μM) (Sigma–Aldrich, U.S.A.) or LY294002 + miR-26a-5p mimic. Sequences of miR-26a-5p mimic, mimic NC, miR-26a-5p inhibitor, and inhibitor NC are listed as follow: miR-26a-5p mimic sense: 5′-UUCAAGUAAUCCAGGAUAGGCU-3′, anti-sense: 5′-CCUAUCCUGGAUUACUUGAAUU-3′. mimic NC: sense: 5′-UUCUCCGAACGUGUCACGUTT-3′, anti-sense: 5′-ACGUGACACGUUCGGAGAATT -3′. MiR-26a-5p inhibitor: 5′-AGCCUAUCCUGGAUUACUUGAA-3′. Inhibitor NC: 5′-CAGUACU UUUGUGUAGUACAA-3′.

### Dual luciferase reporter assay

The wild-type PTEN 3′UTR and mutated PTEN 3′UTR were cloned into psiCHECK-2 vector (Promega, U.S.A.) to construct psiCHECK-2-PTEN-W 3′UTR (wild type) and psiCHECK-2-PTEN-M 3′UTR (mutant) according to manufacturer’s instruction. MiR-26a-5p mimic (or mimic NC) and the luciferase vector were co-transfected into RA-FLS when RA-FLS were grown to 60–80% of confluence in a 24-well plate. Cells were collected 48 h later for fluorescence detection. Both renilla luciferase activity and firefly luciferase activity were measured, and firefly luciferase activity was used as a control for normalization.

### Western blot

MiR-26a-5p transfection and LY294002 treatment were done as previously described. Total protein of RA-FLS was collected by using a Membrane and Cytosol Protein Extraction kit (Beyotime, China). The cell proteins were separated by using SDS/PAGE. The proteins in gel were transferred into a small wet PVDF membrane, followed by blocked with a blocking buffer (Tris-buffered saline, 0.1% Tween 20, and 5% skimmed milks). Then the membrane was incubated with primary antibody of PTEN, GAPDH, β-actin, AKT, phosphorylated AKT (p-AKT Ser473) (CST, U.S.A.), and goat anti-rabbit secondary antibody (Beyotime, China). Intensity of protein bands was evaluated by quantity 1D analysis software and gel doc imaging system, using PTEN as a standard.

### Cells proliferation assay

RA-FLS proliferation was assessed by cell counting-kit-8 (CCK-8) (beyotime, China) assay. MiR-26a-5p mimic, inhibitor or NC was transfected into RA-FLS as previously described. Cells proliferation assay was performed from day 0 (before administration) to day 4 (after administration) following the manufacturer’s instruction. The optical density was measured at 450 nm using a microplate reader (Thermo Scientific, U.S.A.), and all the tests were performed independently in triplicate.

### Cells cycle assay

Cells cycle assay was assessed by LSR Ⅱflow cytometry (BD, U.S.A.). RA-FLS was treated with miR-26a-5p mimic, inhibitor or NC as previously described. After incubation for 48 h, cells were harvested and fixed with 70% ice-cold anhydrous ethyl alcohol overnight at 4°C. Then the cells were washed with PBS and incubated with 500 μl PBS (50 μg/ml propidium, 0.2% Triton X-100, 100 μg/ml RNase A). The specific fluorescence intensity was detected by fluorescence-label flow cytometry.

### Apoptosis analysis

Apoptosis analysis was also assessed with Annexin V-FITC/propidium iodide apoptosis detection kit (Sigma–Aldrich, U.S.A.) using LSR II flow cytometry (BD, U.S.A.). Cells were plated in a 48-well plate at a density of 0.5 × 10^6^ per well. After incubation for 48 h post-transfection, cells were harvested and incubated with 10 μl Annexin V-FITC and 5 μl PI Staining Solution. Flow cytometry data were analyzed using CellQuestPro software (BD, U.S.A.).

### Cells invasion assay

RA-FLS invasion ability was assessed by using transwell 24-well chambers (Corning, U.S.A.). MiR-26a-5p mimic, inhibitor or NC was transfected into RA-FLS as previously described. RA-FLS (2 × 10^3^/well) were seeded in the upper chamber coated with Matrigel, while the lower chamber was filled with 500 μl DMEM medium containing 10% FBS. After incubation for 24 h, cells that invaded the gel and Matrigel to the lower chamber of membrane were fixed with 4% paraformaldehyde for the count of cells for 30 min. Then, the membranes were dried and cells that penetrated to the bottom were stained with crystal violet (Beyotime, China) for 20 min and counted.

### Statistical analysis

The continuous data were described as means ± S.D., and the categorical data were described as numbers (n). Comparison of independent samples was performed by using student’s *t* test, One-way ANOVA or non-parametric test (Wilcoxon rank sum test) using SPSS 21.0 software (IBM Corp, Armonk, NY, U.S.A.). The figures were created using Photoshop 7.0 software (GraphPad Software, U.S.A.). A value of *P*<0.05 was considered to be statistically significant.

## Results

### Up-regulated expression of miR-26a-5p was observed in RA-FLS

**Figure 1:**
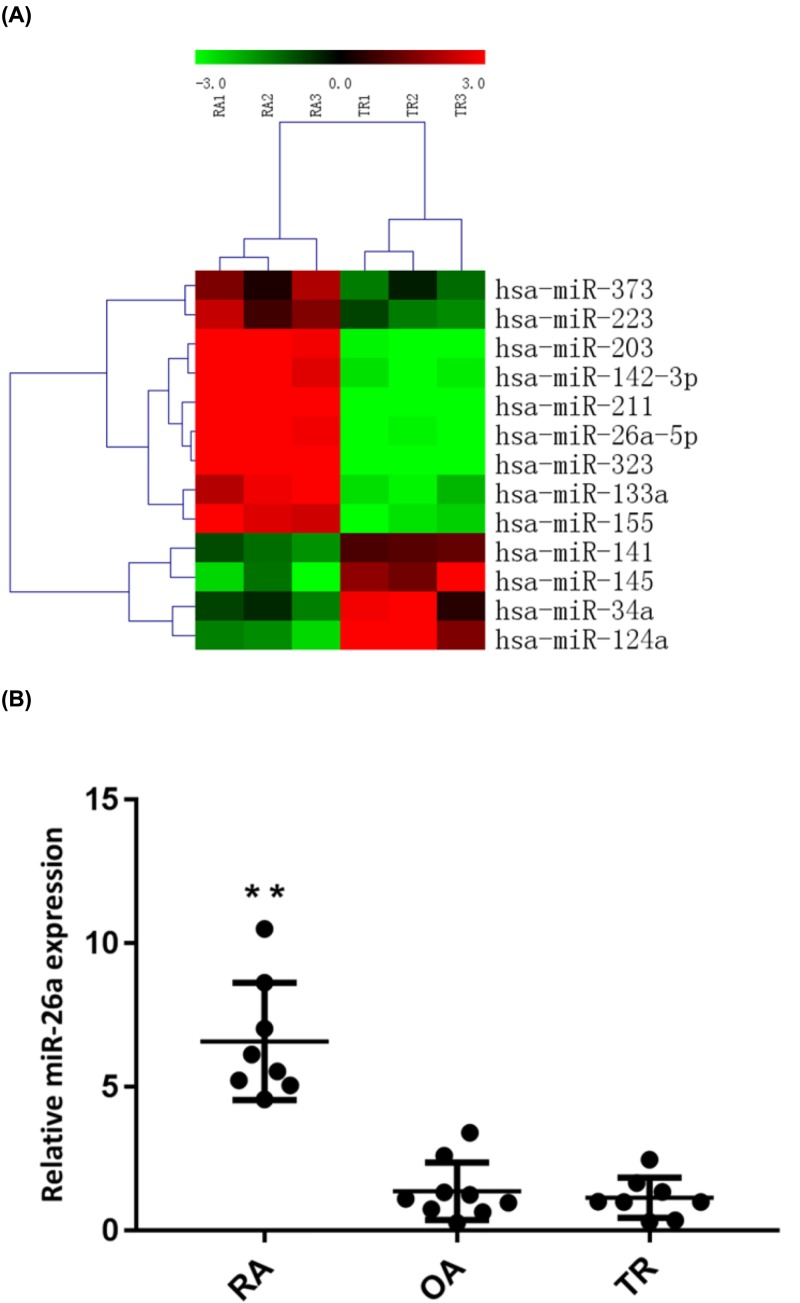
Up-regulated expression of miR-26a-5p was found in RA-FLS by miRNA microarray analysis and confirmed by quantitative RT-PCR analysis **(A)** The altered expression profile of miRNAs in RA-FLS compared with trauma FLS (control) by miRNA microarray analysis. (Red: up-regulation; green: down-regulation) **(B)** Compared with OA (n = 9) and trauma (n = 8), levels of miR-26a-5p is up-regulated in RA-FLS (n = 8), determined by quantitative RT-PCR analysis. **P<0.01.

The expression profiles of miRNAs in fibroblast-like synoviocytes from three RA patients and three knee joint trauma patients were evaluated by miRNA microarray analysis. Compared with fibroblast-like synoviocytes from knee joint trauma patients, up-regulated expression of nine miRNAs (miR-203, miR-323, miR-223, miR-142-3p, miR-133a, miR-211, miR-26a-5p, miR-373, miR-155) were observed in RA-FLS, whereas four miRNAs (miR-141, miR-34a, miR-145, miR-124a) were significantly down-regulated (Figure 1A). To further verify the altered expression of miR-26a-5p from microarray data, quantitative RT-PCR analysis were performed in FLS samples from eight RA patients, eight osteoarthritis (OA) patients and nine knee joint trauma patients. It suggested that levels of miR-26a-5p was 5.1-fold higher in cultured RA-FLS than in OAFLS and 6.1-fold higher than in fibroblast-like synoviocytes from knee trauma patients (*P*<0.01) (Figure 1B).

### MiR-26a-5p promoted cells proliferation in RA-FLS

**Figure 2:**
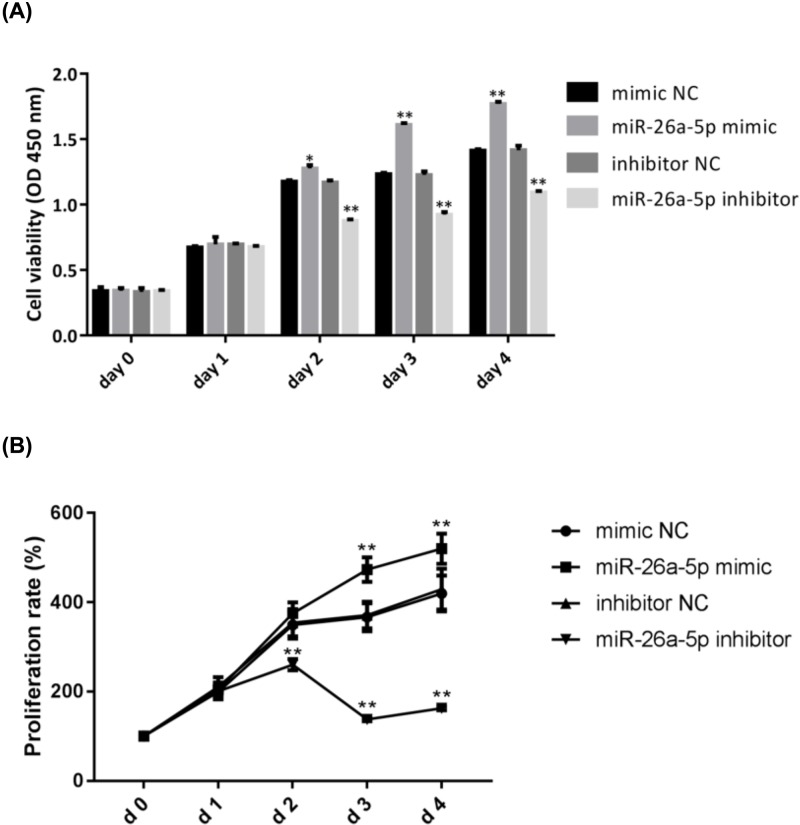
Effect of miR-26a-5p on cells proliferation in RA-FLS **(A)** CCK8 analysis showed that miR-26a-5p mimic promoted cell proliferation in RA-FLS and miR-26a-5p inhibitor had an inhibitory effect on cell proliferation. **(B)** Cell proliferation rate in RA-FLS significantly increased in miR-26a-5p mimic group and decreased in miR-26a-5p inhibitor group from day 2. (*P<0.05, **P<0.01).

The effect of miR-26a-5p on cells proliferation in RA-FLS was determined from day 0 (before administration) to day 4 (after administration) using CCK8 assays. MiR-26a-5p mimic highly stimulated the growth of RA-FLS from day 2 (Figure 2A). Reversely, miR-26a-5p inhibitor inhibited the growth of RA-FLS from day 2 (Figure 2A). Results showed that cell proliferation rate was slightly higher in RA-FLS transfected with miR-26a-5p mimic compared with that transfected with mimic NC in day 2 (375.30 ± 24.59% vs 349.33 ± 30.42%, *P*<0.05), and was obviously higher in RA-FLS transfected with miR-26a-5p mimic compared with that transfected with mimic NC in day 4 (520.06 ± 33.70% vs 419.88 ± 40.05%, *P*<0.01) (Figure 2B). On the contrary, CCK8 assays demonstrated that down-regulation of miR-26a-5p exerted an inhibitory effect on cells proliferation in RA-FLS (Figure 2B).

### MiR-26a-5p promoted G1/S transition in RA-FLS

**Figure 3:**
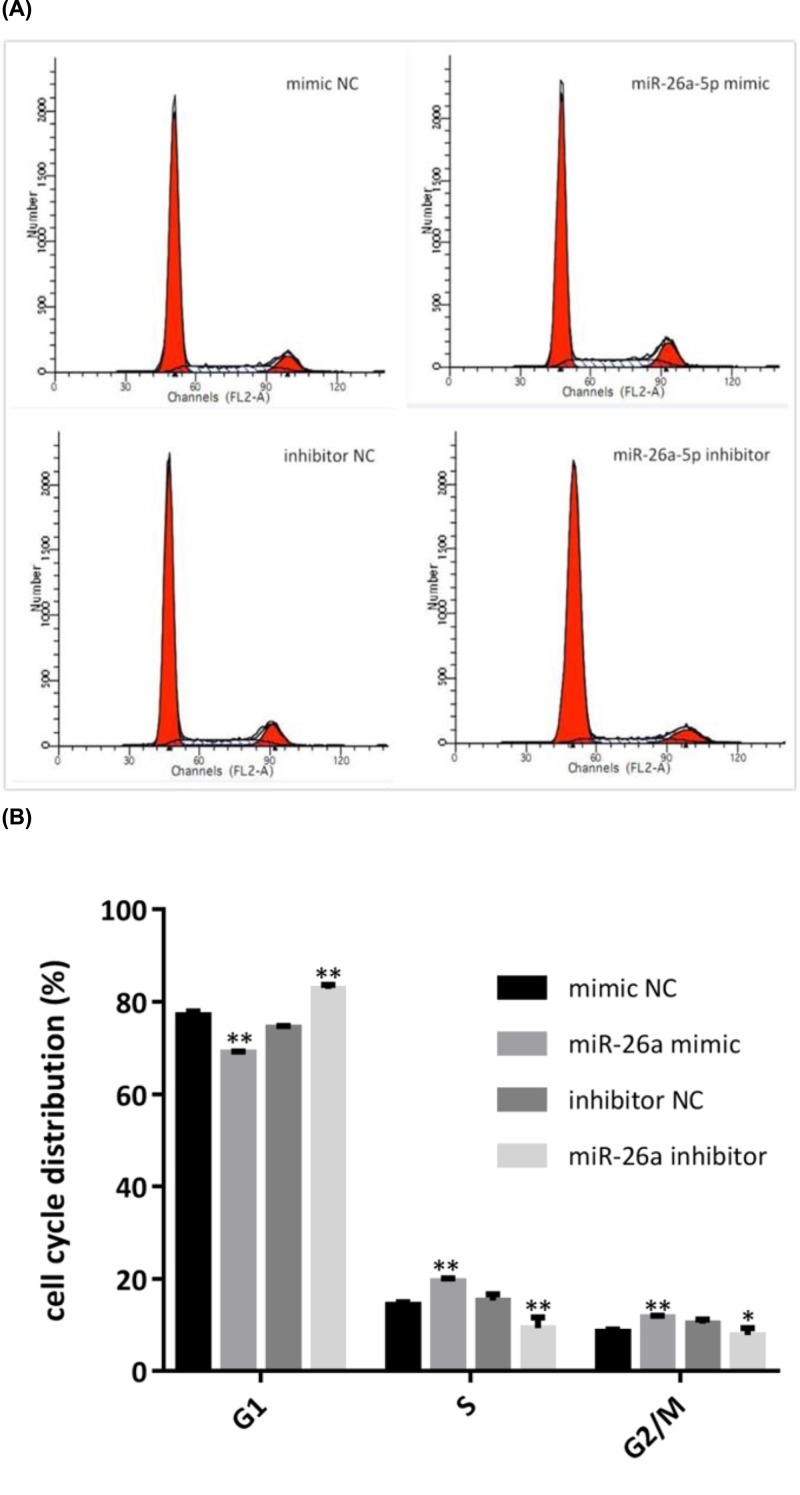
MiR-26a-5p promoted G1/S transition in RA-FLS by cell cycle analysis **(A)** Distribution of cell cycle at different phases, measured by flow cytometry analysis. **(B)** The cell percentages at different phases indicated a cell cycle acceleration in G1/S transition when treated with miR-26a-5p mimic, while a cell cycle deceleration in G1/S transition when treated with miR-26a-5p inhibitor (*P<0.05, **P<0.01).

Flow cytometry indicated altered distribution of cell cycle at different phases in RA-FLS when regulating the expression of miR-26a-5p (Figure 3A). Briefly, cells at the G1 phase significantly decreased in miR-26a-5p mimic-transfected RA-FLS compared with mimic NC (68.95 ± 0.35 vs 77.05 ± 0.88, *P*<0.01), followed by increased in S phase (19.4 ± 0.73 vs 14.39 ± 0.55, *P*<0.01) and G2/M phase (11.65 ± 0.38 vs 8.56 ± 0.49, *P*<0.01) (Figure 3B). However, when treated with miR-26a-5p inhibitor, cells at the G1 phase significantly increased in miR-26a-5p mimic-transfected RA-FLS compared with mimic NC (82.84 ± 0.88 vs 74.39 ± 0.45, *P*<0.01), followed by decreased in S phase (9.31 ± 2.33 vs 15.3 ± 1.36, *P*<0.01) and G2/M phase (7.86 ± 1.46 vs 10.31 ± 0.91, *P*<0.05) (Figure 3B).

### MiR-26a-5p inhibited cells apoptosis in RA-FLS

**Figure 4:**
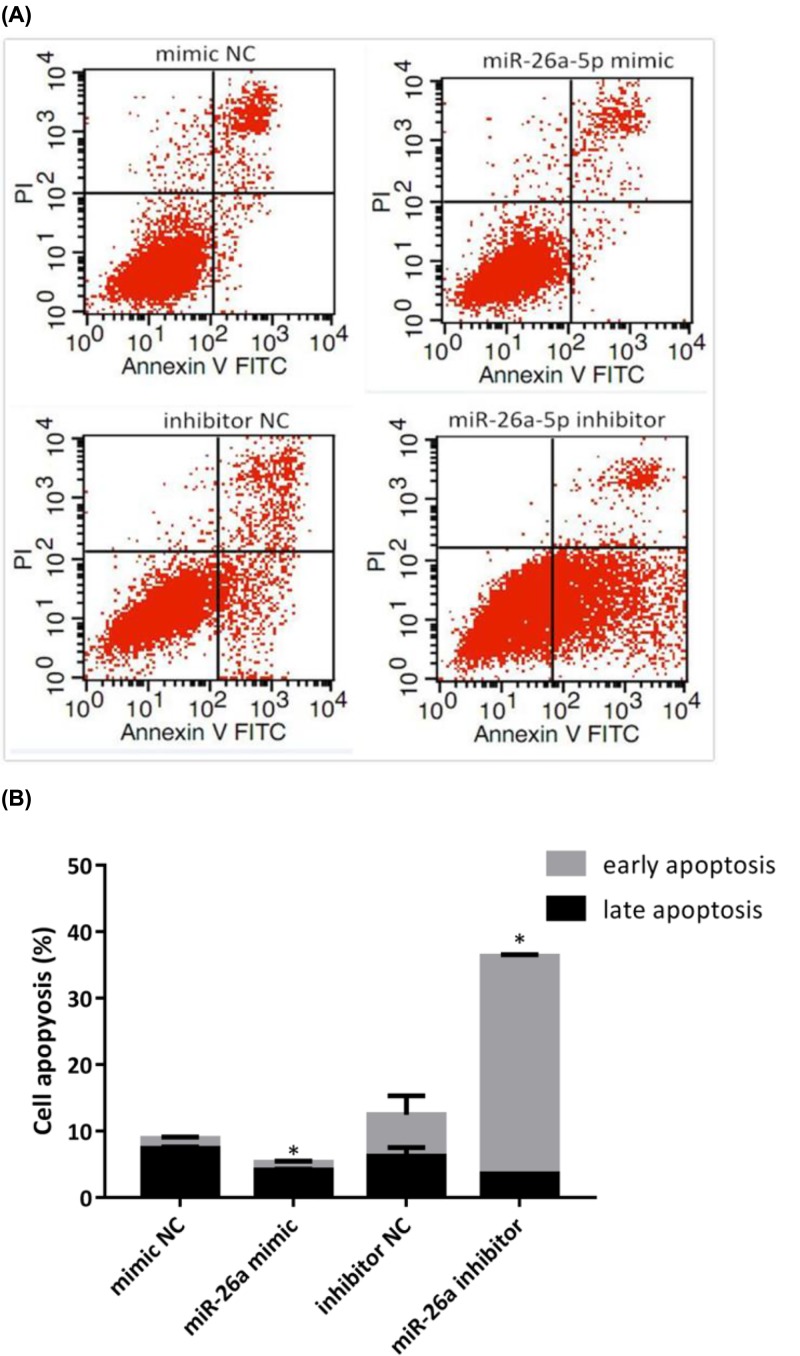
MiR-26a-5p prohibited cell apoptosis in RA-FLS **(A)** Annexin V-FITC/PI assay was used to measure cell apoptosis in RA-FLS. (**B**) Late apoptosis rate reduced in RA-FLS treated with miR-26a-5p mimic when compared with that treated with mimic NC; both early and late apoptosis rate increased RA-FLS treated with miR-26a-5p inhibitor when compared with that treated with inhibitor NC. (*P<0.05).

After treated with miR-26a-5p mimic, inhibitor or NC, cells apoptosis was appraised in RA-FLS using flow cytometry (Figure 4A). Results showed that the percentage of late apoptosis rate reduced in RA-FLS treated with miR-26a-5p mimic when compared with that treated with mimic NC (4.09 ± 0.2 vs 7.37 ± 0.25, *P*<0.05), when early apoptosis rate indicated no difference between the two groups (Figure 4B). Early apoptosis rate (32.85 ± 0.2 vs 6.21 ± 2.88, *P*<0.01) increased in RA-FLS treated with miR-26a-5p inhibitor when compared with that treated with inhibitor NC, whereas late apoptosis rate reduced in RA-FLS treated with miR-26a-5p mimic when compared with that treated with inhibitor NC (3.51 ± 0.06 vs 6.21 ± 1.32, *P*<0.01) (Figure 4B). However, miR-26a-5p inhibitor significantly induced the overall apoptosis including early and late apoptosis in RA-FLS compared with inhibitor NC (*P*<0.01) (Figure 4B).

### MiR-26a-5p enhanced cells invasion in RA-FLS

**Figure 5:**
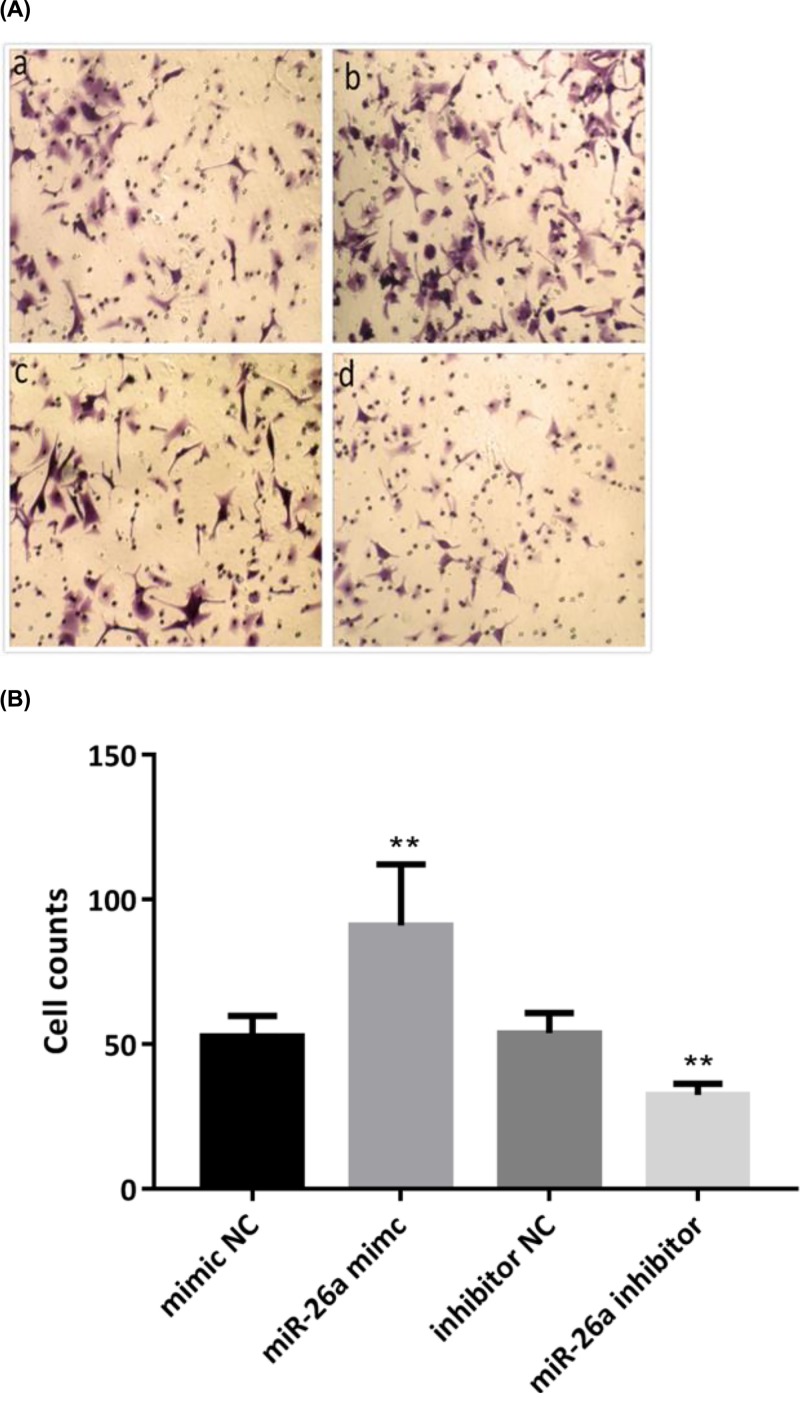
MiR-26a-5p promoted cells invasion in RA-FLS **(A)** More cells invaded the gel and matrigel to the lower chamber of membrane when treated with miR-26a mimic. **(B)** Number of RA-FLS invaded after 24 h is presented. (**P<0.01).

Effect of miR-26a-5p on the cells invasion ability of RA-FLS was determined by Transwell matrigel invasion assays after incubation for 24 h with miR-26a-5p mimic, inhibitor or NC (Figure 5). Results showed that overexpression of miR-26a-5p strengthen the cells invasion in RA-FLS, while down-regulation of miR-26a-5p inhibited cell invasion. More cells invaded the gel and Matrigel to the lower chamber of membrane in RA-FLS treated with miR-26a-5p mimic, when compare with that treated with mimic NC (94 ± 20.28 vs 51.5±6.98, *P*<0.01). Reversely, miR-26a-5p inhibitor decreased cells invasion when compare with that treated with inhibitor NC (54.5 ± 6.5 vs 33.17 ± 3.97, *P*<0.01).

### MiR-26a-5p directly targets PTEN

**Figure 6:**
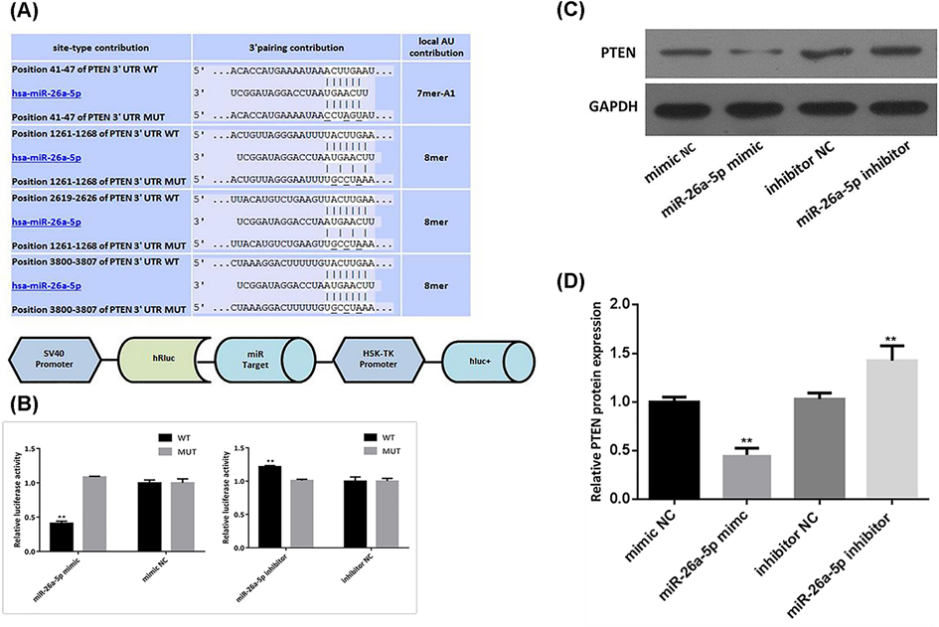
MiR-26a-5p attenuated PTEN expressions **(A)** The predicted region of PTEN 3′UTR targetted by miR-26a-5p (predicted by TargetScanHuman 7.1). Nucleotide changes for binding site mutants are indicated. And the schematic presentation of the reporter plasmid used to illustrate the effect of PTEN 3′UTR on luciferase activity. **(B)** PTEN was the directed targetted gene of miR-26a-5p, confirmed by the luciferase reporter system. **(C)** MiR-26a-5p suppressed the expression of PTEN protein, measure by western blot. (*P<0.05, **P<0.01).

To further investigate the underlying mechanism of miR-26a-5p in RA-FLS, TargetScan (http://www.targetscan.org/vert_72/), microRNA.org (http://www.microrna.org/microrna/home.do) and PicTar (https://pictar.mdc-berlin.de/) were employed to predict the potential targets of miR-26a-5p. PTEN, an important regulator for cells growth and function, was predicted to be a potential target of miR-26a-5p by bioinformatics analysis. Using TargetScan, it was found that four putative miR-26a-5p seed match sites targets in the 3′UTR of PTEN (Figure 6A). To validate whether miR-26a-5p can directly target PTEN, a dual luciferase report gene system was constructed (Figure 6A). Overexpression of miR-26a-5p significantly suppressed the luciferase activity of psiCHECK-2-PTEN-W 3′UTR in RA-FLS, whereas had no effect on the luciferase activity of psiCHECK-2-PTEN-M 3′UTR (Figure 6B). Western blot to further confirm the effect of miR-26a-5p on PTEN was performed. It suggested that miR-26a-5p mimic significantly decreased PTEN expression while miR-26a-5p inhibitor significantly up-regulate expression of PTEN in RA-FLS (Figure 6C).

### MiR-26a-5p mediates the activation of PI3K-AKT pathway

**Figure 7:**
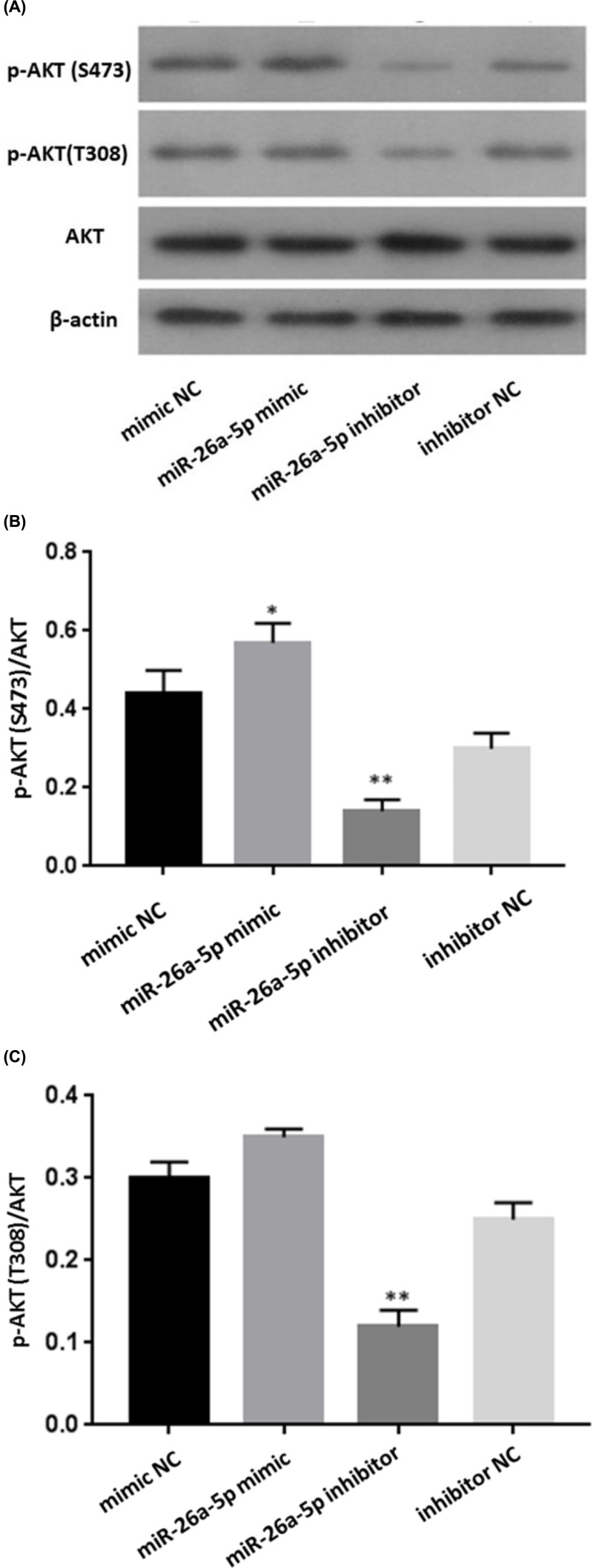
miR-26a-5p regulated protein expression of p-AKT **(A)** The expressions of PI3K/AKT pathway relevant proteins (AKT and p-AKT) after transfection. **(B,C)** p-AKT (S473)/AKT ratio in RA-FLS transfected with miR-26a-5p mimic was significantly higher than that transfected with mimic control, and p-AKT (both T308 and S473)/AKT ratio in RA-FLS transfected with miR-26a-5p inhibitor was significantly lower than that transfected with inhibitor control. (*P<0.05, **P<0.01).

**Figure 8:**
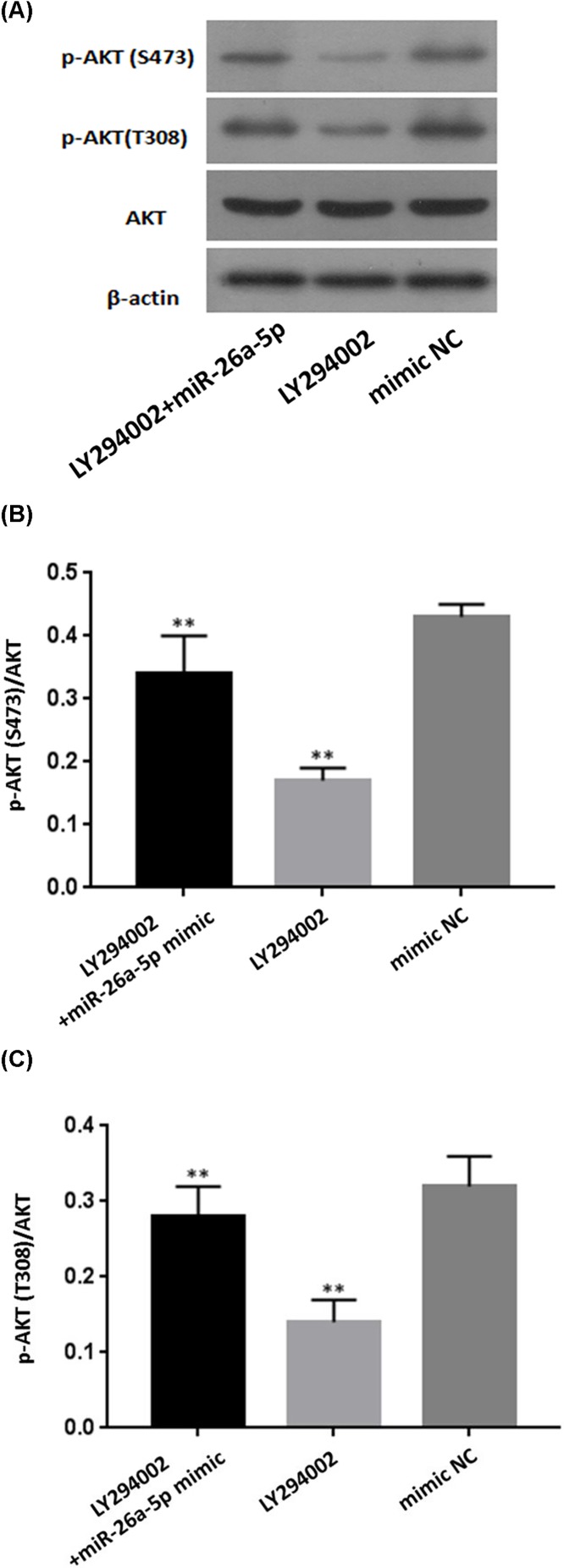
MiR-26a-5p reversed the inhibitory effect of LY294002 on PI3K/AKT pathway **(A)** The expressions of PI3K/AKT pathway relevant proteins (AKT and p-AKT) after transfection and LY294002 treatment. **(B,C)** p-AKT (both S473 and T308)/AKT ratio in RA-FLS transfected with LY294002 was significantly lower than that transfected with mimic control, and p-AKT (both S473 and T308)/AKT ratio in RA-FLS transfected with both LY294002 and miR-26a-5p mimic was significantly higher than that transfected with LY294002. (**P<0.01).

To clarify whether miR-26a-5p promoted the activation of PI3K-AKT pathway in RA-FLS, protein expression of AKT and p-AKT levels were analyzed in cell lysates by western blotting at 48 h after transfection with miR-26a-5p mimic, mimic NC, miR-26a-5p inhibitor, and inhibitor NC. It was shown that overexpression of miR-26a-5p by transfected with miR-26a-5p mimic up-regulated protein expression of p-AKT, while no change was observed regarding to protein expression of total AKT, despite the presence of miR-26a-5p (Figure 7). Densitometry results showed that the p-AKT(S473)/AKT ratio in RA-FLS transfected with miR-26a-5p mimic was significantly higher than that transfected with mimic control (*P*<0.05). Reversely, protein expression of p-AKT was inhibited by miR-26a-5p inhibitor, while in RA-FLS transfected with miR-26a-5p inhibitor, while protein expression of total AKT remained unchanged in RA-FLS transfected with miR-26a-5p inhibitor. Densitometry results showed that the p-AKT (both S473 and T308)/AKT ratio in RA-FLS transfected with miR-26a-5p inhibitor was significantly lower than that transfected with inhibitor control (*P*<0.01). In addition, RA-FLS cells were treated with the PI3K/Akt inhibitor LY294002 or LY294002 + miR-26a-5p mimic (Figure 8). p-AKT (both S473 and T308)/AKT ratio in RA-FLS transfected with LY294002 was significantly lower than that transfected with mimic control (*P*<0.01), and p-AKT (both T308 and S473)/AKT ratio in RA-FLS transfected with both LY294002 and miR-26a-5p mimic was significantly higher than that transfected with LY294002 (*P*<0.01). Thus, miR-26a-5p reversed the inhibitory effect of LY294002 on PI3K/AKT pathway.

## Discussion

RA-FLS, contributing to the formation of hyperplastic synovial pannus tissue, are one of the key effector cells in the pathogenesis of rheumatoid arthritis [23]. RA-FLS are linked to the initiation, perpetuation, and progression of RA by producing pro-inflammatory cytokines and a variety of cell adhesion molecule and protein kinases, inducing inflammation and finally leading to destruction of cartilage and bone [24]. According to previous studies, a group of miRNAs have been found to be dysregulated in RA-FLS, including miR-133a, miR-142-3p, miR-142-5p, miR-146a, miR-155, miR-203, miR-323-3p, miR-124a, and miR-34a [25]. Several miRNAs were demonstrated to be involved in a series of the fundamental biologic processes in RA by regulating RA-FLS proliferation, invasion, apoptosis, and cell secretion [18,26–28]. By preliminary screening with microarray analysis and confirmation with qRT PCR, we identified miR-26a-5p as a new miRNA which was up-regulated in RA-FLS.

It has been revealed that miR-26a-5p plays multiple and converse roles in proliferation and metastasis of different cancers via regulation of different targets. Several studies suggested that miR-26a-5p acts as a suppressor in cancer tissues [29,30]. Significantly growth inhibition was found in estrogen stimulated tumor xenograft models and ER+ breast cancer cells when up-regulating miR-26a expression [30]. Moreover, some researches demonstrated miR-26a-5p may also indirectly promote initiation and progression of some cancers [20,31,32]. Up-regulated expression of miR-26a was observed in gastric cancer cells MKN-28 and promoted cells proliferation, migration and invasion [32]. miR-26a-5p was found to be significantly increased in plasma and tissue from bladder cancer tissues and promoted the progression of bladder cancer [20].

Apart from regulation roles in cancers, miR-26a-5p also has important roles in the regulation of cells function in non-cancer diseases [33,34]. It was found that miR-26a was up-regulated during skeletal muscle differentiation and overexpression of miR-26a promoted myoblasts differentiation while inhibition of miR-26a by regulating Smad1 and Smad4 [33]. To better investigate the effect of miR-26a-5p on RA-FLS proliferation, we assessed cell cycle progression and found that overexpression of miR-26a-5p highly stimulated the growth of RA-FLS from day 2 along with reduction of G1 phase regarding to distribution of cell cycle. Reversely, cell proliferation rate in RA-FLS transfected with miR-26a-5p inhibitor reached its peak in day 2, indicated an inhibitory effect on cell proliferation when down-regulated miR-26a-5p expression. Taken together, our results suggested that miR-26a-5p promotes cell cycle progression and proliferation of RA-FLS. Overexpression of miR-26a-5p also reduced apoptosis rate in RA-FLS while inhibition of miR-26a-5p induced the overall apoptosis. Furthermore, a much larger amount of cells invaded the gel and Matrigel to the lower chamber of membrane in RA-FLS when overexpressed miR-26a-5p. Hence, our data showed that overexpression of miR-26a-5p strengthened cells proliferation, invasion, and apoptosis resistance in RA-FLS, while miR-26a-5p was down-regulated along with the attenuation of cells proliferation, invasion, and apoptosis resistance.

It is well known that PTEN is a common and important tumor suppressor involved in multiple types of cancers via regulating downstream signal pathways [35–37]. Mutations or deletions of PTEN were observed in a variety of tumors [38–40]. Therefore, regulation of PTEN may have some potential effects in RA-FLS, which is known to exhibit several tumor cell-like characteristics. In fact, a lack of PETN expression has been found in the lining layer of RA synovial tissue, which might be contributed to the invasive behavior of RA-FLS [41].

PI3K/AKT signal pathway is a common and central outgrowth and survival pathway, which regulated cell biological functions in various diseases [42,43]. As one of key regulators in this pathway, PTEN dephosphorylates PIP3 to PIP2, which leads to suppression of PI3K/Akt signaling pathway, whereas inhibition of PTEN promotes the activation of the PI3K/Akt pathway [44]. Similarly, our study revealed that PTEN was a direct target of miR-26a-5p and PTEN expression was significantly negative correlated with miR-26a-5p expression in RA-FLS. miR-26a-5p had activation effect on PI3K/AKT signaling pathway via targetting PTEN. Hence, our study supported that miR-26a-5p is an inhibitory factor of PTEN and the effect of miR-26a-5p on cells proliferation, cells invasion and apoptosis resistance in RA-FLS may be associated with activation of PI3K/AKT signaling pathway via targetting PTEN.

## Conclusion

**Figure 9:**
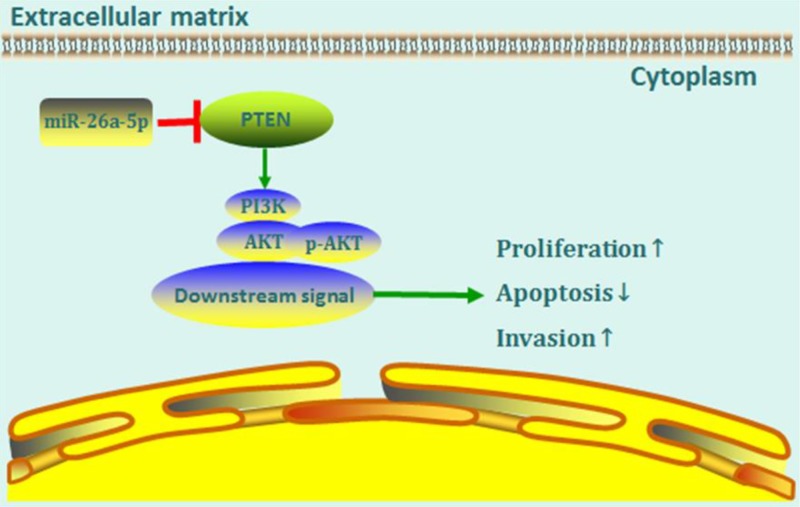
A proposed model for miR-26a-5p-mediated mechanism in RA-FLS MiR-26a-5p, by targetting PTEN, enhances cells proliferation, invasion as well as apoptosis resistance of RA-FLS via PI3K/AKT pathwa.

In summary, we identified miR-26a-5p as a new miRNA that is up-regulated in RA-FLS. Our study suggests that overexpression of miR-26a-5p RA-FLS promoted cells proliferation, cells invasion, and apoptosis resistance in RA-FLS. Furthermore, we find that the biological effects of miR-26a-5p on RA-FLS, at least partially, may be attributed to its activation effect on PI3K/AKT signaling pathway via targetting PTEN. In conclusion, our study implies that up-regulated miR-26a-5p in RA-FLS is possibly involved in synovial tissue pathological changes in RA, and miR-26a-5p may serve as a new therapeutic target in RA (Figure 9).

## Supporting information

**Supplementary Table S1:** Demographic and clinical data of included participants

## Abbreviations

AKT: protein kinase B
CCK-8: cell counting-kit-8
FBS: fetal bovine serum
FLS: fibroblast-like synoviocyte
GAPDH: glyceraldehyde-3-phosphate dehydrogenase
NC: negative control
OA: osteoarthritis
PBS: phosphate-buffered saline
PTEN: gene of phosphate and tension homology deleted on chromsome ten
RA: rheumatoid arthritis

## References

[1] Scott D.L., Wolfe F. and Huizinga T.W. (2010) Rheumatoid arthritis. Lancet (London, England) 376, 1094–1108 10.1016/S0140-6736(10)60826-4 20870100

[2] Assayag D., Lee J.S. and King T.E. Jr (2014) Rheumatoid arthritis associated interstitial lung disease: a review. Medicina (B. Aires) 74, 158–165 24736263

[3] Huber L.C., Distler O., Tarner I., Gay R.E., Gay S. and Pap T. (2006) Synovial fibroblasts: key players in rheumatoid arthritis. Rheumatology (Oxford) 45, 669–675 10.1093/rheumatology/kel065 16567358

[4] Mor A., Abramson S.B. and Pillinger M.H. (2005) The fibroblast-like synovial cell in rheumatoid arthritis: a key player in inflammation and joint destruction. Clin. Immunol. 115, 118–28 10.1016/j.clim.2004.12.009 15885632

[5] Turner J.D. and Filer A. (2015) The role of the synovial fibroblast in rheumatoid arthritis pathogenesis. Curr. Opin. Rheumatol. 27, 175–182 10.1097/BOR.0000000000000148 25603041

[6] Calabresi E., Petrelli F., Bonifacio A.F., Puxeddu I. and Alunno A. (2018) One year in review 2018: pathogenesis of rheumatoid arthritis. Clin. Exp. Rheumatol. 36, 175–184 29716677

[7] Bartel D.P. (2009) MicroRNAs: target recognition and regulatory functions. Cell 136, 215–233 10.1016/j.cell.2009.01.002 19167326 PMC3794896

[8] Bader A.G., Brown D. and Winkler M. (2010) The promise of microRNA replacement therapy. Cancer Res. 70, 7027–7030 10.1158/0008-5472.CAN-10-2010 20807816 PMC2940943

[9] Lerner M., Lundgren J., Akhoondi S., Jahn A., Ng H.F., Akbari Moqadam F. (2011) MiRNA-27a controls FBW7/hCDC4-dependent cyclin E degradation and cell cycle progression. Cell Cycle 10, 2172–2183 10.4161/cc.10.13.16248 21597324

[10] Lu K., Wang J., Song Y., Zhao S., Liu H., Tang D. (2015) miRNA-24-3p promotes cell proliferation and inhibits apoptosis in human breast cancer by targeting p27Kip1. Oncol. Rep. 34, 995–1002 10.3892/or.2015.4025 26044523

[11] Lu L.F. and Liston A. (2009) MicroRNA in the immune system, microRNA as an immune system. Immunology 127, 291–298 10.1111/j.1365-2567.2009.03092.x 19538248 PMC2712098

[12] Rupaimoole R. and Slack F.J. (2017) MicroRNA therapeutics: towards a new era for the management of cancer and other diseases. Nat. Rev. Drug Discov. 16, 203–222 10.1038/nrd.2016.246 28209991

[13] Dalal S.R. and Kwon J.H. (2010) The role of microRNA in inflammatory bowel disease. Gastroenterol. Hepatol. 6, 714–722 PMC3033542 21437020

[14] Cao R.Y., Li Q., Miao Y., Zhang Y., Yuan W., Fan L. (2016) The emerging role of microRNA-155 in cardiovascular diseases, Biomed Res Med. 2016, 1–5 28018919 10.1155/2016/9869208 PMC5149600

[15] Rasmussen T.K., Andersen T., Bak R.O., Yiu G., Sorensen C.M., Stengaard-Pedersen K. (2015) Overexpression of microRNA-155 increases IL-21 mediated STAT3 signaling and IL-21 production in systemic lupus erythematosus. Arthritis Res. Ther. 17, 154 26055806 10.1186/s13075-015-0660-z PMC4504038

[16] Rajasekhar M., Olsson A.M., Steel K.J., Georgouli M., Ranasinghe U., Brender Read C. (2017) MicroRNA-155 contributes to enhanced resistance to apoptosis in monocytes from patients with rheumatoid arthritis. J. Autoimmun. 79, 53–62 10.1016/j.jaut.2017.01.002 28118944 PMC5397583

[17] Trenkmann M., Brock M., Gay R.E., Michel B.A., Gay S. and Huber L.C. (2013) Tumor necrosis factor alpha-induced microRNA-18a activates rheumatoid arthritis synovial fibroblasts through a feedback loop in NF-kappaB signaling. Arthritis Rheum. 65, 916–927 10.1002/art.37834 23280137

[18] Stanczyk J., Ospelt C., Karouzakis E., Filer A., Raza K., Kolling C. (2011) Altered expression of microRNA-203 in rheumatoid arthritis synovial fibroblasts and its role in fibroblast activation. Arthritis Rheum. 63, 373–381 10.1002/art.30115 21279994 PMC3116142

[19] Song Q., Liu B., Li X., Zhang Q., Cao L., Xu M. (2018) MiR-26a-5p potentiates metastasis of human lung cancer cells by regulating ITGbeta8- JAK2/STAT3 axis. Biochem. Biophys. Res. Commun. 501, 494–500 10.1016/j.bbrc.2018.05.020 29746867

[20] Wang H., Hu Z. and Chen L. (2018) Enhanced plasma miR-26a-5p promotes the progression of bladder cancer via targeting PTEN. Oncol. Lett. 16, 4223–4228 30197668 10.3892/ol.2018.9163 PMC6126335

[21] Ma J., Fan Y., Zhang J., Feng S., Hu Z., Qiu W. (2018) Testosterone-dependent miR-26a-5p and let-7g-5p Act as signaling mediators to regulate sperm apoptosis via targeting PTEN and PMAIP1. Int. J. Mol. Sci. 19, 10.3390/ijms19041233 PMC5979296 29670053

[22] Aletaha D., Neogi T., Silman A.J., Funovits J., Felson D.T., Bingham C.O. 3rd (2010) 2010 rheumatoid arthritis classification criteria: an American College of Rheumatology/European League Against Rheumatism collaborative initiative. Ann. Rheum. Dis. 69, 1580–1588 10.1136/ard.2010.138461 20699241

[23] Bartok B. and Firestein G.S. (2010) Fibroblast-like synoviocytes: key effector cells in rheumatoid arthritis. Immunol. Rev. 233, 233–255 10.1111/j.0105-2896.2009.00859.x 20193003 PMC2913689

[24] Bottini N. and Firestein G.S. (2013) Duality of fibroblast-like synoviocytes in RA: passive responders and imprinted aggressors. Nat. Rev. Rheumatol. 9, 24–33 10.1038/nrrheum.2012.190 23147896 PMC3970924

[25] Churov A.V., Oleinik E.K. and Knip M. (2015) MicroRNAs in rheumatoid arthritis: altered expression and diagnostic potential. Autoimmun. Rev. 14, 1029–1037 10.1016/j.autrev.2015.07.005 26164649

[26] Yang S. and Yang Y. (2015) Downregulation of microRNA221 decreases migration and invasion in fibroblastlike synoviocytes in rheumatoid arthritis. Mol. Med. Rep. 12, 2395–2401 10.3892/mmr.2015.3642 25891943

[27] Li X.F., Shen W.W., Sun Y.Y., Li W.X., Sun Z.H., Liu Y.H. (2016) MicroRNA-20a negatively regulates expression of NLRP3-inflammasome by targeting TXNIP in adjuvant-induced arthritis fibroblast-like synoviocytes. Joint Bone Spine 83, 695–700 10.1016/j.jbspin.2015.10.007 26934991

[28] Gao J., Zhou X.L., Kong R.N., Ji L.M., He L.L. and Zhao D.B. (2016) microRNA-126 targeting PIK3R2 promotes rheumatoid arthritis synovial fibro-blasts proliferation and resistance to apoptosis by regulating PI3K/AKT pathway. Exp. Mol. Pathol. 100, 192–198 10.1016/j.yexmp.2015.12.015 26723864

[29] Miyamoto K., Seki N., Matsushita R., Yonemori M., Yoshino H., Nakagawa M. (2016) Tumour-suppressive miRNA-26a-5p and miR-26b-5p inhibit cell aggressiveness by regulating PLOD2 in bladder cancer. Br. J. Cancer 115, 354–363 10.1038/bjc.2016.179 27310702 PMC4973152

[30] Tan S., Ding K., Li R., Zhang W., Li G., Kong X. (2014) Identification of miR-26 as a key mediator of estrogen stimulated cell proliferation by targeting CHD1, GREB1 and KPNA2. Breast Cancer Res. 16, R40 10.1186/bcr3644 24735615 PMC4053242

[31] Huse J.T., Brennan C., Hambardzumyan D., Wee B., Pena J., Rouhanifard S.H. (2009) The PTEN-regulating microRNA miR-26a is amplified in high-grade glioma and facilitates gliomagenesis in vivo. Genes Dev. 23, 1327–1337 19487573 10.1101/gad.1777409 PMC2701585

[32] Ding K., Wu Z., Wang N., Wang X., Wang Y., Qian P. (2017) MiR-26a performs converse roles in proliferation and metastasis of different gastric cancer cells via regulating of PTEN expression. Pathol. Res. Pract. 213, 467–475 10.1016/j.prp.2017.01.026 28242043

[33] Dey B.K., Gagan J., Yan Z. and Dutta A. (2012) miR-26a is required for skeletal muscle differentiation and regeneration in mice. Genes Dev. 26, 2180–2191 23028144 10.1101/gad.198085.112 PMC3465739

[34] Chen L., Zeng W., Yang B., Cui X., Feng C., Wang L. (2017) Expression of antisense of microRNA-26a-5p in mesenchymal stem cells increases their therapeutic effects against cirrhosis. Am. J. Transl. Res. 9, 1500–1508 28386375 PMC5376040

[35] Sansal I. and Sellers W.R. (2004) The biology and clinical relevance of the PTEN tumor suppressor pathway. J. Clin. Oncol. 22, 2954–2963 10.1200/JCO.2004.02.141 15254063

[36] Lee Y.R., Chen M. and Pandolfi P.P. (2018) The functions and regulation of the PTEN tumour suppressor: new modes and prospects. Nat. Rev. Mol. Cell Biol. 19, 547–562 10.1038/s41580-018-0015-0 29858604

[37] Song M.S., Salmena L. and Pandolfi P.P. (2012) The functions and regulation of the PTEN tumour suppressor. Nat. Rev. Mol. Cell Biol. 13, 283–296 10.1038/nrm3330 22473468

[38] Perren A., Komminoth P., Saremaslani P., Matter C., Feurer S., Lees J.A. (2000) Mutation and expression analyses reveal differential subcellular compartmentalization of PTEN in endocrine pancreatic tumors compared to normal islet cells. Am. J. Pathol. 157, 1097–1103 10.1016/S0002-9440(10)64624-X 11021813 PMC1850183

[39] Negoro K., Takahashi S., Kinouchi Y., Takagi S., Hiwatashi N., Ichinohasama R. (2000) Analysis of the PTEN gene mutation in polyposis syndromes and sporadic gastrointestinal tumors in Japanese patients. Dis. Colon Rectum 43, S29–S33 10.1007/BF02237223 11052475

[40] Halachmi N., Halachmi S., Evron E., Cairns P., Okami K., Saji M. (1998) Somatic mutations of the PTEN tumor suppressor gene in sporadic follicular thyroid tumors. Genes, Chromosomes Cancer 23, 239–243 9790504 10.1002/(sici)1098-2264(199811)23:3<239::aid-gcc5>3.0.co;2-2

[41] Kinne R.W., Palombo-Kinne E. and Emmrich F. (1995) Activation of synovial fibroblasts in rheumatoid arthritis. Ann. Rheum. Dis. 54, 501–504 10.1136/ard.54.6.501-b 7632096 PMC1009912

[42] Juntilla M.M. and Koretzky G.A. (2008) Critical roles of the PI3K/Akt signaling pathway in T cell development. Immunol. Lett. 116, 104–110 10.1016/j.imlet.2007.12.008 18243340 PMC2322870

[43] Martini M., De Santis M.C., Braccini L., Gulluni F. and Hirsch E. (2014) PI3K/AKT signaling pathway and cancer: an updated review. Ann. Med. 46, 372–383 10.3109/07853890.2014.912836 24897931

[44] Wyatt L.A., Filbin M.T. and Keirstead H.S. (2014) PTEN inhibition enhances neurite outgrowth in human embryonic stem cell-derived neuronal progenitor cells. J. Comp. Neurol. 522, 2741–2755 10.1002/cne.23580 24610700

