# Supplementary material for: MiR-26a-5p enhances cells proliferation, invasion, and apoptosis resistance of fibroblast-like synoviocytes in rheumatoid arthritis by regulating PTEN/PI3K/AKT pathway
Source: Biosci Rep. 2019 Jul 26;39(7):BSR20182192. doi: 10.1042/BSR20182192 (PMC6658817; doi:10.1042/BSR20182192)
Supplement: Supplementary file 1 [file bsr20182192_Supp1.pdf]

Supplementary Table 1. Demographic and clinical data of included participants

Groups	Name	Gender	Age (Y)	Disease Duration	Position	Date
RA	XB Xiao	Male	45	13 years	Right Knee	2018.3.30
RA	ZK Yang	Male	61	16 years	Right Knee	2018.4.3
RA	YS He	Female	77	15 years	Right Knee	2018.4.22
RA	Sy Chen	Male	60	23 years	Right Knee	2018.5.14
RA	Hh Lv	Female	53	21 years	Left Knee	2018.5.14
RA	RH Lin	Female	63	17 years	Left Knee	2018.4.3
RA	YW Zheng	Female	51	11 years	Right Knee	2018.6.1
RA	QX Li	Female	50	16 years	Left Knee	2018.6.6
Trauma	GH Huang	Male	43	10 days	Right Knee	2018.4.5
Trauma	YX Zhang	Female	35	7 days	Right Knee	2018.4.11
Trauma	HQ W	Female	55	1 days	Right Knee	2018.4.15
Trauma	GH Sheng	Male	88	1 days	Left Knee	2018.4.19
Trauma	ZY Li	Female	47	1 days	Left Knee	2018.4.22
Trauma	XL Li	Male	29	1 days	Left Knee	2018.5.4
Trauma	JL Wu	Female	56	32 days	Left Knee	2018.5.13
Trauma	SH Wu	Male	55	2 days	Right Knee	2018.5.16
Trauma	GY Liang	Male	72	20 days	Left Knee	2018.5.21
OA	JY Lai	Female	47	13 years	Right Knee	2018.3.28
OA	HC Cai	Male	52	21 years	Right Knee	2018.4.7
OA	JW Huang	Female	61	15 years	Left Knee	2018.4.7
OA	Y Wang	Female	71	33 years	Left Knee	2018.4.18
OA	YH Mo	Female	56	12 years	Right Knee	2018.5.7
OA	XQ Chen	Female	61	30 years	Left Knee	2018.5.12
OA	HH Hong	Male	55	10 years	Right Knee	2018.5.12
OA	ZK Liu	Female	68	32 years	Left Knee	2018.5.25
